# Challenges and hope: latest research trends in the clinical treatment and prognosis of liposarcoma

**DOI:** 10.3389/fphar.2025.1529755

**Published:** 2025-05-12

**Authors:** Hongliang Liu, Xi Wang, Xiaoyu Wang, Fabo Qiu, Bin Zhou

**Affiliations:** ^1^ Department of Hepatobiliary and Pancreatic Surgery and Retroperitoneal Tumor Surgery, The Affiliated Hospital of Qingdao University, Qingdao, China; ^2^ Department of Oncology, Women and Children’s Hospital Affiliated to Qingdao University, Qingdao, China; ^3^ Department of Anesthesiology Department, The Affiliated Hospital of Qingdao University, Qingdao, China

**Keywords:** liposarcoma, clinical and molecular characteristics, surgery, systemic treatment, prognosis

## Abstract

Liposarcoma, as a complex disease, is characterized by intricate interactions between distinct histopathological subtypes and corresponding clinical outcomes, emphasizing the necessity of personalized approaches in diagnosis and treatment strategies. This malignant tumor originating from adipose tissue is classified into different subtypes with specific molecular markers, which not only distinguish them but also guide treatment directions. The main approach for treating liposarcoma is surgical resection, with the aim of complete excision and achieving clean margins (R0 resection) to minimize the risk of recurrence. This surgical principle emphasizes the critical need for precise preoperative planning, and in certain cases, the integration of neoadjuvant therapy may be needed to reduce the tumor to a surgically manageable size. In addition to surgery, systemic therapy plays a key role in the advanced stages of the disease, especially when resistance to traditional treatment arises. The emergence of novel systemic therapies, including chemotherapy, targeted therapy, and immunotherapy, has opened new avenues for treating this challenging malignancy. These systemic therapies are selected on the basis of the specific molecular features of the tumor, highlighting the importance of detailed molecular diagnostics. As our understanding of the molecular basis of liposarcoma deepens, integrating clinical and molecular features is crucial for optimizing treatment outcomes. This comprehensive approach, which combines surgical precision with systemic therapy innovations, will change the treatment landscape for patients with liposarcoma, advancing toward more personalized and effective treatment strategies.

## 1 Introduction

Soft tissue sarcomas (STSs) account for 1% of adult malignancies and are a group of mesenchymal tumors comprising 179 histological subtypes (https://seer.cancer.gov/statfacts/html/soft.html) (Kallen and Hornick, 2021). Liposarcoma (LPS) accounts for 15%–20% of STSs and is a rare malignant tumor characterized by adipocyte differentiation (Gronchi et al., 2021). Approximately 41% of LPSs occur in the lower limbs, 36% in the retroperitoneum, 8% in the upper limbs, and 5% each in visceral organs and the trunk (De vita et al., 2016). According to the 5th edition of the WHO Classification of Soft Tissue Tumors released in 2020 (Schaefer and Gronchi, 2022), subtypes of LPS include atypical lipomatous tumor (ALT)/well-differentiated liposarcoma (WDLPS), dedifferentiated liposarcoma (DDLPS), myxoid liposarcoma (MLPS), pleomorphic liposarcoma (PLPS), and myxoid pleomorphic liposarcoma (MPLPS), with MPLPS being a new addition characterized by a nonspecific nuclear pattern, lacking the classic gene fusion of DDIT3 with FUS or EWSR1. Given that these subtypes have unique clinical, histological, biological, immunohistochemical, and molecular genetic features relevant to diagnosis, prognosis, and treatment sensitivity (Kallen and Hornick, 2021), individualized treatment methods should be formulated on the basis of the histological type (Haddox and Riedel, 2020). A series of novel antitumor drugs that target the specific molecular biology of LPS are actively being researched, offering hope for increasing treatment options for recurrent or unresectable LPS (Lee et al., 2018).

## 2 Clinical and molecular characteristics of each subtype of LPS

Each subtype of liposarcoma (LPS) has unique clinical and molecular characteristics, reflecting the diversity and complexity of this malignant tumor (Yao et al., 2020).

### 2.1 Cytogenetic characteristics of ALT/WDLPS and DDLPS

**FIGURE 1:**
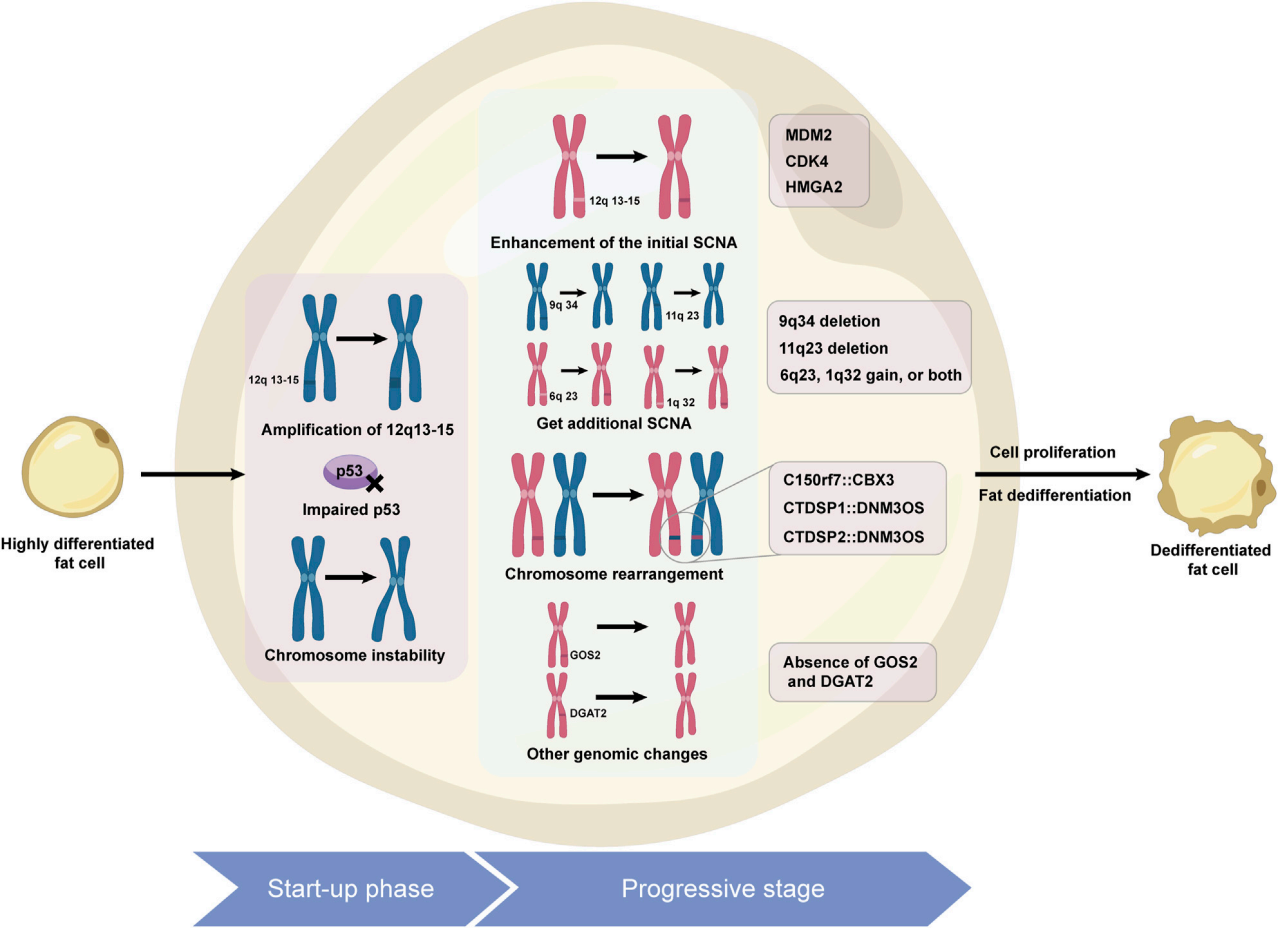
Molecular mechanisms driving dedifferentiation in liposarcoma.

ALT/WDLPS is the most common subtype of liposarcoma, accounting for 40%–45%, and is commonly found in the limbs, buttocks, and deep soft tissues of the trunk, with 25% originating from the retroperitoneum. Its subtypes include lipomatous, sclerosing, inflammatory, and spindle cell variants (Sciot et al., 2020). They usually grow slowly, are prone to recurrence, and are resistant to radiotherapy and chemotherapy. Compared with WDLPS of the limbs, retroperitoneal WDLPS has a greater risk of dedifferentiation (Keung et al., 2018). DDLPS accounts for 15%–20% of cases, mostly in middle-aged and elderly people, with 75% occurring in the abdominal cavity and retroperitoneum (Gahvari and Parkes, 2020; Kilpatrick, 2021). DDLPS is characterized by increased aggressiveness and metastatic potential and is chemoresistant (Ghadimi et al., 2011). On a pathological level, it may exhibit homologous and heterologous dedifferentiation, with the majority being of high grade (Schaefer and Gronchi, 2022). DDLPS has a local recurrence rate of up to 40%, with a distant metastasis rate of 15%–30%, and the site of the lesion is an important prognostic factor. The molecular hallmark of WDLPS and DDLPS is amplification of chromosomal region 12q13-15 (Matthews et al., 2010), particularly amplification of the MDM2 and CDK4 genes (Aleixo et al., 2009), which drive tumor growth and dedifferentiation. Other key genes in this region, such as HMGA2, TSPAN31, FRS2, and GLI1, and new genes outside this area, such as DDR2, SDHC, and FGFR, also play significant roles in its pathogenesis (Pentimalli et al., 2003; Saâda-bouzid et al., 2015; Gao et al., 2013; Kanojia et al., 2015; Barretina et al., 2010). The signaling pathways of the FGFR/FRS2 and the PIK3R3/ERK/Nanog axis are closely linked to the development of DDLPS (Wang et al., 2011; Zhang et al., 2013; Jing et al., 2018). While DDLPS shares common cytogenetic features, it presents more genomic abnormalities and complexity, exerting a greater impact on treatment response and prognosis. Mechanisms of liposarcoma dedifferentiation can be seen in Figure 1.

### 2.2 The genetic characteristics of MLPS

MLPS is the predominant form of LPS among children and adolescents, accounting for 20%–30% of cases, and is found mainly in the deep soft tissue of the limbs, particularly near the proximal thigh, with a rare occurrence in the retroperitoneum (approximately 2.3%) (Henze and Bauer, 2013). Approximately 12%–25% of patients are likely to experience local recurrence, and between 30% and 60% can metastasize (Schaefer and Gronchi, 2022). Pathology findings have shown that the presence of round cell components is associated with a poor prognosis (Schaefer and Gronchi, 2022; Setsu et al., 2016). In patients with MLS, dedifferentiation is rare (Ciongariu et al., 2023). More than 95% of MLS patients present with a t (12; 16) (q13; p11) translocation, leading to the production of the FUS-DDIT3 fusion protein, impeding adipocyte terminal differentiation and facilitating tumor formation (Pérez-mancera et al., 2008; Powers et al., 2010; Xiao et al., 2018). Next-generation sequencing has identified new fusion genes and signaling pathway abnormalities, such as RET, FGFR2 (Künstlinger et al., 2015; Napolitano et al., 2021), PI3K/AKT/mTOR (Trautmann et al., 2019a; Berthold et al., 2022), Hippo/YAP1 dysregulation (Regina and Hettmer, 2019; Trautmann et al., 2019b), and TERT promoter mutations (Koelsche et al., 2014), revealing that FUS-CHOP activates the SRC/FAK/RHO/ROCK signaling axis, enhancing the invasive capacity of MLS cells (Tornin et al., 2018). Staaberg’s team reported that a subgroup of MLS cells with CSC characteristics activate the JAK-STAT signaling pathway (Dolatabadi et al., 2019), which could be a significant target for MLS treatment.

### 2.3 Genetic alterations in PLPS and MPLPS

PLPS, a subtype characterized by high invasiveness and poor prognosis, accounts for just 5%–10% of all LPSs, featuring elevated rates of local recurrence and metastasis (approximately 30%–50% each), with a 5-year survival rate of 60% (Schaefer and Gronchi, 2022). It affects mainly the deep soft tissues of the limbs, particularly the lower extremities. There is significant chemoresistance, which may be associated with P53 mutations. PLPS is characterized by pronounced chromosomal abnormalities, encompassing deletions and duplications (Conyers et al., 2011). Some studies indicate a correlation between RB1 mutations and PLPS (Libbrecht et al., 2021). MPLPS is a highly aggressive, rare tumor that predominantly occurs in the mediastinum of children and young adults and affects mainly females. It has complex chromosomal alterations and lacks FUS-DDIT3 gene fusion and MDM2/CDK4 gene amplification (Schaefer and Gronchi, 2022).

The diversity of the LPS subtypes in terms of clinical manifestations and molecular pathology underscores the need for a meticulous approach in diagnosis, treatment, and research to accommodate the unique characteristics of each subtype. Advances in molecular genetics offer promising avenues for targeted treatments, emphasizing the importance of continued research to fully leverage these outcomes to improve patient care.

## 3 LPS treatment

### 3.1 Surgical intervention

The primary treatment for LPS is to perform R0 surgical resection as much as possible, avoiding unplanned resection (Qureshi et al., 2012). The survival duration of primary localized RPL is relatively short, with an overall 8-year survival rate ranging from 30% to 80% for different subtypes (Siew et al., 2022). All patients with resectable RPL should undergo initial extensive surgical resection for complete R0 excision (Delisle et al., 2022; Harati et al., 2017). Research has shown a significant association between OS, DFS, the LR rate, and LRFS in RPLPS patients and R0 resection (Paik et al., 2022). Compartment resection is the current standard procedure. Precision surgical principles require surgical stratification on the basis of the biological behavior of RPL. For radiotherapy and chemotherapy-resistant LPS, such as WDLPS/DDLPS, surgical intervention remains the cornerstone of treatment. In case of surgical difficulties, neoadjuvant chemotherapy or radiotherapy may be considered to reduce the risk of recurrence. Given the high recurrence rate of RPL, measures such as radiotherapy and drug therapy may be considered, but controversy remains (Rust et al., 2022; D’ambrosio et al., 2022). Surgical intervention may have a certain effect on locally recurrent RPL, but the likelihood of long-term control decreases after each recurrence (Tseng et al., 2022). Research by Maria Anna Smolle et al. revealed that the survival rate of patients with primary localized limb MLPS who undergo metastatic liver resection after recurrence is higher than that of those who receive other treatments (Smolle et al., 2020). Studies suggest that simultaneous resection of the primary tumor and metastases in patients with LPS presenting with distant metastasis at diagnosis may prolong survival (Illuminati et al., 2010). Multiple studies have demonstrated that metastasectomy can increase survival rates (Chudgar et al., 2017; Marudanayagam et al., 2011). The Japanese JCO guidelines recommend resection of the primary lesion and metastases, but further research is needed on patient selection, considering factors such as patient condition, number of metastases, and status of the primary tumor. Studies (Tirotta et al., 2020) by Tirotta F et al. suggested that LRSM can result in prolonged patient survival, although factors such as extrahepatic metastases, large metastatic lesions, chemotherapy resistance, and short DFI contribute to reduced survival rates.

### 3.2 Chemotherapeutic treatment

#### 3.2.1 Neoadjuvant chemotherapy for high-risk patients with resectable LPS

For patients at very high risk or with early resection difficulties, neoadjuvant chemotherapy regimens resemble those of advanced treatment, frequently employing anthracycline-based agents. Meta-analyses demonstrated a 6% reduction in mortality risk with perioperative chemotherapy and an 11% reduction with the standard A + I regimen (Woll et al., 2012). Phase III trials have shown that the A + I regimen is superior to trabectedin (Gronchi et al., 2017; Gronchi et al., 2020). The results of phase II trials are promising (Tanaka et al., 2015; Tanaka et al., 2019). There is no evidence supporting the use of neoadjuvant chemotherapy for resectable RPS (Gronchi et al., 2021; Swallow et al., 2021). The TARPSWG study recommended the adoption of the A + I regimen for Grade 3 DDLPS patients (Tseng et al., 2021). The STRASS2 trial evaluated histology-tailored neoadjuvant chemotherapy, with the DDLPS regimen being doxorubicin + ifosfamide (Istl and Gronchi, 2022).

#### 3.2.2 Systemic therapy for unresectable, advanced, or metastatic LPS

The efficacy of chemotherapy and overall survival rates vary depending on the LPS subtype (Schöffski, 2022). MLP exhibits high sensitivity to chemotherapy (Hindi and Haas, 2022); PLPS shows relative sensitivity to chemotherapy (Italiano et al., 2012); DDLPS demonstrates some response, whereas ALT/WDLPS are generally insensitive to chemotherapy. The median overall survival (mOS) for chemotherapy-sensitive subtypes in advanced stages is approximately 2 years (Abbas Manji et al., 2015). Presently, the first-line systemic therapy regimen is D + IFO (Crago and Dickson, 2016); however, there are inadequate specific research data on the various subtypes of LPS. Stacchiotti et al. reported that WD/DDLPS patients had response rates of 6.3% and 13% to first-line anthracycline and ifosfamide chemotherapy, respectively, whereas in the D + IFO group, the response rate was 22%. The response rate to first-line chemotherapy is significantly greater in MLPS patients than in WDLPS/DDLPS patients (48% vs. 11%) (Jones et al., 2005), and the overall survival with first-line chemotherapy is significantly longer in MPLS patients than in DDLPS/PLPS patients (Langmans et al., 2019).

Second-line and subsequent regimens include high-dose continuous infusion of ifosfamide, gemcitabine-based combination therapy (such as docetaxel and dacarbazine), and novel chemotherapy agents, including trabectedin, eribulin, and dacarbazine (Chamberlain et al., 2021). Second-line treatment with trabectedin significantly prolongs progression-free survival (PFS) in advanced LPS patients (Patel et al., 2019; Le cesne et al., 2021; De sande gonzález et al., 2020; Vincenzi et al., 2023). MLS is more sensitive to trabectedin (Assi et al., 2019). For unresectable/recurrent STS patients, the overall median PFS is 3.7 months, with a median PFS of 17.4 months for MLS patients and 3.7 months for DDLPS patients (Kobayashi et al., 2020). Trabectedin can be safely administered to elderly STS patients who are unsuitable for first-line anthracycline therapy (Grosso et al., 2020). Eribulin affects tumor cells and the microenvironment by inhibiting microtubule growth and through various molecular mechanisms (De vita et al., 2021), with chemotherapy-induced peripheral neuropathy (CIPN) being the principal adverse effect. Phase III trials indicate that eribulin monotherapy is superior to dacarbazine monotherapy in patients with locally advanced, recurrent, or metastatic LPS (Frapolli et al., 2019). Novel treatment strategies, such as the combination of eribulin with lenvatinib and eribulin combined with gemcitabine, show promising efficacy (Chen et al., 2022; Kim et al., 2022). Future directions involve enhancing efficacy, mitigating toxicity, and identifying biomarkers to predict treatment response. Other novel agents, such as cabazitaxel, exhibit favorable activity in advanced DDLPS (Sanfilippo et al., 2022). Ascorbic acid and carfilzomib also demonstrate potential therapeutic effects (Schoenfeld et al., 2018; Jeitany et al., 2021).

#### 3.2.3 Others

Research by Miao Chengli et al. revealed that performing HIPEC after surgery for retroperitoneal LPS can significantly reduce mortality and recurrence rates (Miao et al., 2022). Angeles et al. discovered that SN-38 induces apoptosis in DDLPS cells by increasing C/EBPα protein expression (Angeles et al., 2022).

### 3.3 Targeted therapy

#### 3.3.1 Targeting MDM2

Currently, the MDM2 inhibitor DS-3032b shows potential efficacy in patients with WDLPS/DDLPS (Bauer et al., 2018), with comparative trials underway (Gounder et al., 2022a). The efficacy of AMG 232 is also under investigation (Gluck et al., 2020), and the MANTRA trial revealed that milademetan has failed as a second-line treatment for unresectable or metastatic DDLPS patients (Jones et al., 2023). Brigimadlin has demonstrated potential antitumor activity in DDLPS/WDLPS (Lorusso et al., 2023), with global phase II/III studies currently underway. Studies by Cissé MY et al. reported that MDM2-mediated serine metabolism control is a driving force in the growth of LPS (Cissé et al., 2020), whereas Seligson ND et al. suggested that targeting HDAC2 may be a potential strategy for modulating MDM2 expression in DDLPS (Seligson et al., 2019).

#### 3.3.2 Targeting CDK4

In WD/DDLPS, the amplification rate of CDK4 is as high as 90%, making CDK4 another viable target (Assi et al., 2020). The CSCO guidelines recommend palbociclib as a second-line treatment, but practical application studies show poor outcomes (Nassif et al., 2022). The combination of palbociclib with recombinant methionase enhanced the efficacy of palbociclib (Higuchi et al., 2022). A phase III study of abemaciclib versus placebo is underway. MDM2 inhibitors combined with CDK4/6 inhibitors show manageable toxicity and good antitumor activity in advanced-stage patients (Abdul Razak et al., 2022).

#### 3.3.3 Targeting vascular endothelial growth factor

Studies have shown that LPS contains more microvessels (Baneth et al., 2005) and is more sensitive to antiangiogenic therapy. Anlotinib, as a second-line treatment for STS, is included in the CSCO guidelines. The ALTER0202 study showed significant efficacy, with a 12-week PFR of 63% and mPFS and mOS of 5.6 and 13 months, respectively (Chi et al., 2018). The ALTER-S006 study indicated that patients who were maintained on anlotinib after first-line chemotherapy had an mPFS of 12.5 months (Xu et al., 2023). Retrospective studies have shown that treatment with anlotinib in patients with metastatic or recurrent WDLPS/DDLPS resulted in an mPFS of 27.9 weeks, a 24-week PFR of 58.8%, and an OS of 56.6 weeks (Li et al., 2021).

#### 3.3.4 Multitargeted tyrosine kinase inhibitors

Pazopanib is a second-line treatment option for STS recommended by the National Comprehensive Cancer Network (NCCN) guidelines (Cassinelli et al., 2022), but its efficacy as a monotherapy for LPS is limited. Phase II research revealed that pazopanib treatment resulted in a 12-week PFR of 68.3%, and the mPFS for DDLPS patients was 6.24 months (Samuels et al., 2017). A German phase II trial compared the efficacy of pazopanib combined with gemcitabine versus pazopanib alone in treating refractory LPS/LMS patients, noting an increase in toxicity with the combination treatment, which was manageable; however, phase III trials are needed to confirm its efficacy (Schmoll et al., 2021). Another phase II study evaluating preoperative pazopanib in high-risk STS patients reported no benefit (Ronellenfitsch et al., 2019). The SARC024 study indicated that regorafenib has poor efficacy in patients with advanced LPS (Riedel et al., 2020).

#### 3.3.5 Additional potential targets

PARP-1 has emerged as a new therapeutic target for treating LPS (Bertucci et al., 2019). The TOMAS2 study from Italy revealed that the combination of trabectedin and the PARP inhibitor olaparib is effective in the treatment of LPS/LMS (D’Ambrosio et al., 2023). XPO1 represents another potential therapeutic avenue (Gounder et al., 2016), with selinexor demonstrating enhanced tumor responses in retroperitoneal DDLPS-PDXs (Thirasastr and Somaiah, 2022). The SEAL study revealed that the median PFS for advanced DDLPS patients treated with selinexor as second-line therapy was 2.8 months and that CALB1 could serve as a predictive biomarker (Gounder et al., 2022b). Selinexor treatment can reduce the pain rate in late-stage DDLPS patients, with a slower deterioration in quality of life (Gounder et al., 2021). Future endeavors should continue multidisciplinary research to explore novel drug targets and individualized treatment approaches.

### 3.4 Immunotherapy

Multiple clinical trials have explored immunotherapies for STSs, including ICIs, tumor vaccines, immune modulators, and TCR-T-cell therapy. Although STSs are considered “immunologically inert or cold” tumors, recent biomarker studies have shown significant immunoheterogeneity among different subtypes (Moreno tellez et al., 2022; Zhu et al., 2020). Biomarker-driven and tissue subtype-customized immunotherapy holds promise for improving the efficacy of immunotherapy (Roulleaux dugage et al., 2021). Immunotherapy combined with other treatment modalities, such as chemotherapy and radiotherapy, can transform “cold” tumors into “hot” tumors (Rytlewski et al., 2021). Efficacy biomarkers such as TLSs, PD-L1 expression, and the TMB stratify patients to optimize efficacy, design improved clinical trials, and potentially enhance the effectiveness of immunotherapy (Nakata et al., 2021).

#### 3.4.1 Monotherapy immunotherapy

Monotherapy with ICIs has not yet demonstrated definitive clinical benefits, but pembrolizumab has been shown to have antitumor effects in DDLPS-PDX models (Choi et al., 2020). In SARC028, the DCR for advanced STS patients was 18%, with an ORR of 40% for UPS and 20% for LPS (Tawbi et al., 2017; Burgess et al., 2019). In the Alliance A091401 trial, the overall response rate (ORR) of nivolumab monotherapy in metastatic STS patients was merely 5%.

#### 3.4.2 Combination immunotherapy

Immunotherapy is continuously evolving in the field of LPS, with efforts focused on genomic analysis and research into the tumor immune microenvironment to identify additional combination treatment strategies, aiming to improve the effectiveness of immunotherapy in LPS patients.

##### 3.4.2.1 Immunotherapy combined with chemotherapy

In patients with STS, the ORR of combination therapy with pembrolizumab and doxorubicin was 36.7%, with an mPFS and OS of 5.7 months and 17 months, respectively (Livingston et al., 2021). Among DDLPS patients, 1 patient achieved a complete response (CR), 1 patient achieved a partial response (PR), and 2 patients had stable disease (SD). In patients with L-type sarcoma treated with avapritinib combined with trabectedin, among 11 LPS patients, 7 patients achieved the best response of stable disease (SD), and 1 patient achieved disease stability for over 2 years (Wagner et al., 2022).

##### 3.4.2.2 ICIs combined with antiangiogenic targeted therapy

Previous studies have shown that low-grade sarcomas typically exhibit a weak immune response and that antiangiogenic drugs can convert the immune microenvironment from “cold” to “hot,” increasing the sensitivity of the immune microenvironment to immunotherapy (De vita et al., 2016). J. Wu et al.’s retrospective study (Wu et al., 2023) investigated the treatment of L-type sarcomas with carfilzomib in combination with anlotinib and aidiublin. The ORR was 19.4%, and the DCR was 72.2%. Among nonsurgical patients, the mPFS values for LPS and LMS were 5.5 months and 6.2 months, respectively. Research by Zhou et al. revealed the satisfactory efficacy of pembrolizumab combined with anlotinib and paclitaxel in treating STS, with hematologic toxicity associated with paclitaxel being the primary adverse effect (Zhou et al., 2023).

##### 3.4.2.3 Immunotherapy combined with small molecule inhibitors targeting epigenetics

Various subtypes of STSs exhibit defects in DNA damage repair and abnormalities in epigenetic regulation. Although epigenetic drugs can stimulate the immune system, increasing the immunogenicity of tumors, they may still suppress immune responses in the absence of immune checkpoint inhibitors (Keenan et al., 2019; Nacev et al., 2020). Recent research (Starzer et al., 2021) suggests that the DNA methylation characteristics of tumors may serve as markers for the response to PD-1 ICI therapy in sarcomas. Que et al. reported HDAC gene amplification in patients with LPS, and the HDAC inhibitor chidamide increased PD-L1 expression, facilitating tumor regression (Que et al., 2021). Phase II trials have demonstrated that the combination of chidamide and trastuzumab is highly effective in treating STSs and has good tolerability, indicating promising therapeutic potential (Zhang et al., 2023). Ongoing clinical trials of tazemetostat combined with durvalumab for the treatment of STSs (NCT04705818) may offer new hope for patients.

##### 3.4.2.4 Dual immunotherapy

In a phase II trial conducted by MD Anderson (NCT02815995), the efficacy of the PD-L1 monoclonal antibody durvalumab and the CTLA-4 monoclonal antibody tremelimumab in refractory advanced STSs was evaluated (Somaiah et al., 2022). The ORR was 12%, with a 12-week PFS rate of 49%, a median PFS of 2.8 months, and an mOS of 21.6 months. No effects were observed for LPS or the other subtypes.

#### 3.4.3 Others

In addition to ICIs, immunotherapy involving immune cell therapy is also utilized in patients with STSs. CAR-T-cell therapy and TCR-T-cell therapy are still in their early stages and face various challenges. In the SPEARHEAD-1 study, afami-cel was used to treat patients with MRLPS or SS, resulting in 2 cases of CR, 8 cases of PR, and 11 cases of SD out of 25 patients. NY-ESO-1 is one of the most immunogenic TAAs, with a positivity rate of 89%–100% in MRLPS. Phase I/Ib studies of NY-ESO-1 TCR/IL-15 NK cells are currently underway. In recent years, the CMB305 vaccine has also been utilized in STS research, enhancing immune responses to the NY-ESO-1 antigen. A phase II trial (Chawla et al., 2022) evaluating CMB305 in combination with atezolizumab for MLS/SS patients revealed no significant extension of PFS or OS, but some patients exhibited anti-NY-ESO-1 immune responses, with seemingly favorable radiographic responses. IFN-γ alters the TME, increases antigen presentation, reduces T-cell exhaustion, and can convert tumors into “hot” tumors, potentially synergizing with PD-1 antibodies (Zhang et al., 2019). In an IB/II trial conducted by the University of Iowa (Monga et al., 2021), TVEC combined with neoadjuvant radiotherapy for STSs resulted in SD in 66.7% of patients, PR in 1 MLS patient, death due to PD in 2 patients, and pCR in 7 patients (24%), with 2-year PFS and OS rates reaching 57% and 88%, respectively, without postoperative local recurrence.

Emerging therapeutic modalities such as antibody-drug conjugates (ADCs) and oncolytic viruses demonstrate promising therapeutic potential. Initially deployed in hematologic malignancies, ADC-based therapies achieved their first breakthrough in solid tumors with HER2-positive breast cancer. As of 2024, no ADC clinical trials targeting liposarcoma have received regulatory approval (Xi et al., 2024). The leucine-rich repeat-containing protein 15 (LRRC15), overexpressed in sarcoma-associated cancer-associated fibroblasts, has emerged as a compelling anticancer target. LRRC15-directed ADCs may substantially improve clinical outcomes for sarcoma patients (Ray et al., 2022). Preclinical evidence indicates that BB-1701—a novel eribulin-based ADC engineered for HER2 targeting—represents a potential therapeutic advancement for liposarcoma management (Wang et al., 2024). Talimogene laherparepvec (T-VEC), an oncolytic herpes simplex virus type 1, holds the distinction of being the first oncolytic virus approved by the US FDA and European Medicines Agency (Greig, 2016). In a phase IB/II trial involving 30 patients with locally advanced soft tissue sarcoma (STS), preoperative intratumoral T-VEC combined with concurrent external beam radiotherapy (EBRT) demonstrated no treatment-related herpes infections. The 2-year progression-free survival (PFS) and overall survival (OS) rates were 57% and 88%, respectively (Monga et al., 2021). A phase II trial enrolling 20 patients with locally advanced or metastatic sarcoma evaluated T-VEC plus pembrolizumab, yielding an overall objective response rate (ORR) of 35%, with 20% grade 3 treatment-related adverse events (TRAEs) and no grade 4 TRAEs (Kelly et al., 2020). Another phase II study of 39 pretreated advanced sarcoma patients investigated the TNT regimen (T-VEC + trabectedin + nivolumab), reporting an ORR of 7.7%, disease control rate (DCR) of 84.6%, median PFS of 7.8 months, and median OS of 19.3 months (Chawla et al., 2023). Novel combinatorial therapeutic strategies incorporating oncolytic viruses remain under active investigation.

### 3.5 Radiotherapy

Postoperative LPS is prone to recurrence, and radiotherapy can improve local control rates. Therefore, radiotherapy is strongly recommended for patients with high-risk localized recurrence profiles, while therapeutic de-escalation through radiation omission represents a viable strategy for those with low recurrence probability (Salerno, 2022). For most patients, preoperative delivery of radiation therapy is preferred. In patients initially thought to be at low risk for local recurrence and found to have unexpected adverse pathologic features at resection, postoperative radiation therapy is indicated. In select patients who received preoperative ra-diation and have close or positive margins, postoperative boost may be considered (Salerno, 2022).

MLS is sensitive to radiotherapy and is an important target for radiotherapy. Multiple studies have shown that neoadjuvant radiotherapy combined with surgical resection can achieve a 5-year local control rate of 96%–98% (Guadagnolo et al., 2008; Chung et al., 2009; Moreau et al., 2012). Research (Chen et al., 2021) has shown that the interaction between FUS-CHOP and chromatin remodeling complexes regulates sarcoma cell proliferation, explaining the sensitivity of MLS to radiotherapy. Phase II/III trials (Bonvalot et al., 2019) evaluating neoadjuvant radiotherapy combined with NBTXR3 versus radiotherapy alone in advanced STSs have shown a significant increase in the R0 resection rate (81% vs. 66%; P = 0.042). The standard neoadjuvant radiation therapy dose for MLS is 50 Gy/25 fractions. Low-dose radiation therapy may reduce the complications associated with preoperative radiation therapy while maintaining disease control. A phase II trial (Lansu et al., 2021a) revealed that low-dose preoperative radiation therapy (36 Gy) had comparable efficacy in nonmetastatic MLS and could reduce complications. Another phase II trial (Lansu et al., 2021b) showed that moderate-dose preoperative radiation therapy (36 Gy) could improve the resectability of MLS while preserving clear margins and function.

Radiation therapy is also under investigation for RLPS. The STRASS phase III study (Lam et al., 2021) demonstrated that neoadjuvant radiation therapy reduced the risk of local recurrence in patients with resectable RLPS, with a 3-year ARFS rate of 71.6%. The TARPSWG study (Haas et al., 2019) enrolled 607 RLPS patients, and univariate analysis revealed that perioperative radiation therapy had local control advantages in all three cohorts, but no survival benefit was confirmed after adjustment. An analysis of 2082 RLPS patients from the American Cancer Database revealed that neoadjuvant radiation therapy conferred survival benefits, with a mOS of 129.2 months vs. 84.3 months, with more pronounced effects in those with involvement of adjacent organs. Multidisciplinary discussions are recommended to formulate initial treatment plans, and the selective use of RT may be considered for those at high risk of local recurrence (Istl and Gronchi, 2022; Callegaro et al., 2023).

In the radiotherapeutic management of liposarcoma, emerging modalities continue to undergo rigorous investigation. A clinical study validated the safety profile of proton and carbon ion particle therapy for dedifferentiated liposarcoma (DDLPS), demonstrating favorable overall survival (OS) and local control (LC) outcomes (Kubota et al., 2024). A retrospective analysis of stereotactic body radiotherapy (SBRT) in sarcoma pulmonary metastases revealed prolonged disease-free intervals among oligometastatic patients, with a median survival duration of 40.7 months (Lee et al., 2023). Evidence indicates stereotactic ablative radiotherapy (SABR) serves as a viable local control strategy for limited pulmonary oligometastatic disease, exhibiting minimal toxicity (Baumann et al., 2020; Baumann et al., 2016). A multicenter trial evaluating SABR in oligometastatic soft tissue sarcoma (STS) established its therapeutic efficacy and safety profile, with 20% of patients maintaining progression-free status at 2-year follow-up (Franceschini et al., 2024).

### 3.6 Alternative local therapeutic approaches

Emerging locoregional therapeutic modalities including percutaneous radiofrequency ablation (RFA), cryoablation, and high-intensity focused ultrasound (HIFU) are being increasingly utilized in liposarcoma management. A clinical case demonstrated sustained tumor-free survival exceeding 24 months following RFA treatment in a patient with third recurrence of retroperitoneal liposarcoma involving the left psoas muscle (Keil et al., 2008). Koichiro et al. conducted a retrospective multicenter analysis of percutaneous RFA in 52 recurrent bone and soft tissue sarcoma patients, reporting a 1-year overall survival (OS) rate of 73.4% with minimal major complication rate (0.9%), confirming RFA as a safe and effective option for advanced sarcomas (Yamakado et al., 2014).

A retrospective study of percutaneous cryoablation in 141 adults with recurrent/metastatic soft tissue sarcomas documented 217 ablation procedures achieving adequate ice-ball coverage in 82% (204/250) of lesions. The cohort exhibited a 2% complication rate (4/217) with favorable survival outcomes: 89% 1-year OS and 80% 2-year OS (Pal et al., 2024). Another real-world analysis of 67 recurrent/metastatic STS patients undergoing 189 cryoablation procedures for 104 lesions demonstrated an objective response rate (ORR) of 65.38% and disease control rate (DCR) of 86.54%, with survival analysis indicating prognostic improvement (Wu et al., 2021).

In HIFU applications, a study treating 29 lesions in 22 solid tumor patients achieved near-complete MRI-confirmed ablation in liposarcoma cases with symptomatic relief (Orgera et al., 2011). Yu et al. reported 51.8% ORR and 85.2% local control rate in 27 patients with locally unresectable sarcomas undergoing HIFU, with no severe treatment-related complications observed (Yu et al., 2015).

### 3.7 Multidisciplinary discussions

STSs are diverse and rare, and nonspecialist doctors should refrain from diagnosing and treating them. The MDT diagnostic and treatment model is essential in the management of STSs (Kawai et al., 2022).

## 4 Prognosis

### 4.1 Prognostic factor analysis of primary nonmetastatic extremity or trunk liposarcoma

For patients with nonmetastatic extremity or trunk liposarcoma, studies have shown that tumor size and subtype are independently associated with distant metastasis-free survival (DSD) and disease-specific survival (DR), whereas size, subtype, and R1 resection are independently associated with local recurrence (LR) (Bartlett et al., 2021). These findings suggest that the patterns, risks, and timing of postoperative recurrence vary by subtype, which can guide the development of targeted treatment measures for patients.

### 4.2 Prognostic factor analysis of patients with retroperitoneal liposarcoma

Large-scale population-based international cohort studies have consistently identified advanced age as an independent prognostic factor for overall survival (OS) and cancer-specific survival (CSS) in patients with retroperitoneal liposarcoma (Li et al., 2022; Singer et al., 2003). Gender-specific analysis reveals males exhibit inferior survival outcomes compared to females following primary resection of RLPS, particularly in subsets with low-grade histology or undergoing non-radical resection (R1/R2 resections) (Ren et al., 2024). An Asian multicenter cohort study of 211 patients demonstrated independent associations between American Society of Anesthesiologists (ASA) physical status classification, Clavien-Dindo complication grading system, and long-term OS (Zhuang et al., 2021).

Tumor-related characteristics significantly impact prognostic outcomes in retroperitoneal liposarcoma (RLS) patients. Retrospective cohort analysis demonstrates inferior disease-free survival (DFS) in dedifferentiated histology compared to well-differentiated subtypes (Osuna-soto et al., 2021). Histologic subtype emerged as an independent predictor of progression-free survival (PFS) (Singer et al., 2003; Zhuang et al., 2021). The Fédération Nationale des Centres de Lutte Contre le Cancer (FNCLCC) grading system and myogenic differentiation status constitute critical prognostic determinants (Gronchi et al., 2015). Additionally, tumor anatomical location and presence of necrosis may serve as independent pathologic prognostic indicators (Sun et al., 2021). Tumor rupture and major postoperative complications (Dindo-Clavien grade ≥ III) adversely affect overall survival (OS) (Brehat et al., 2023). Patients developing multifocal recurrence exhibit particularly dismal clinical outcomes (Deng et al., 2023a).

Surgical margin characteristics significantly influence oncologic outcomes in retroperitoneal liposarcoma (RLS). The presence of dedifferentiated (DD) components at resection margins correlates with diminished local recurrence-free survival (LRFS) (Dehner et al., 2021). A comparative effectiveness study demonstrated that total (ipsilateral) retroperitoneal lipectomy (TRL), when contrasted with conventional complete resection (CR), confers significant improvements in both recurrence-free survival (RFS) and overall survival (OS) for primary RPLS patients (Gao et al., 2024).

Recurrence patterns critically determine clinical prognosis in RLS management. Patients exhibiting DR patterns demonstrate more favorable survival trajectories compared to those with early multifocal recurrence (Deng et al., 2023b). Homsy. P et al. analyzed 107 RPL patients and reported that 72% experienced LR, whereas 15% experienced DR, indicating more local recurrence and fewer metastases (Homsy et al., 2020). After R0/R1 resection, histological type and grade were important predictors of DSS, with multifocal LR having a poorer prognosis and a higher DR rate with high-grade histology. Improta. L et al. studied 109 RPL cases, with a 5-year OS rate of 67%, a DFS rate of 53.2%, an LR rate of 25.7%, and a DM rate of 12.1%, with lung metastasis being the most common. Patients with complications had better DFS and OS, and HOI-3 was an independent risk factor for DM, OS, and DFS (Improta et al., 2023). One study reported a 6-year DFS rate of 19.2% and an OS rate of 54.1% for RPS-LR1 patients, with recurrence patterns associated with histological subtypes, and the CCI for the second LR of LPS was the highest (60.2%–70.9%). Column charts predicting DFS and OS were established, incorporating multiple factors (Raut et al., 2019). The TARPSWG study analyzed RPS-R2 patients and reported a 70.5% incidence of second recurrence, with an LR accounting for 80.75%, predominantly LPS (Van houdt et al., 2020). Singaporean scholars proposed a five-gene prognostic model for retroperitoneal DDLPS, which better predicted overall survival than did clinical factors (Shannon et al., 2021).

### 4.3 Potential prognostic significance of immune-related molecular markers and tumor-infiltrating immune cells in LPS

Miyake M et al. reported that PD-L1 expression was higher in retroperitoneal DDLPS and retroperitoneal LMS than in other sarcomas (Miyake et al., 2020). Serum LDH levels were moderately positively correlated with PD-L1 and PD-L2 expression. Higher PD-1 expression was associated with an increased risk of recurrence; High expression of Ki-67 and stage IIIB disease were independent predictors of RFS and DSS. The Ki67 proliferation index has been established as an independent prognostic factor for recurrence, metastasis, and overall survival (OS) in retroperitoneal liposarcoma (RLS) patients undergoing complete resection (Gao et al., 2023). Schroeder BA et al. reported that high TCR clonality combined with a low T-cell fraction predicted a lower 3-year OS rate, that CD4^+^ T cells were associated with better outcomes, and that CD14^+^ monocytes were associated with poorer prognosis (Schroeder et al., 2021). In recent years, research on tertiary lymphoid structures (TLSs) in patients with STSs has increased, with more TLS patients showing longer OS and PFS, associated with increased expression of the TNFRSF14 and DUSP9 genes, and better immunotherapy outcomes (Xiang-Xu et al., 2023). Inflammatory biomarkers such as the NLR or PLR fail to accurately predict survival (Schwartz et al., 2020), with tumor-related factors remaining the best predictors. Kim KM et al. reported that baseline inflammatory markers such as IL-6 were associated with early recurrence of STSs (Van der laan et al., 2023). The nuclear expression of 4Rα and IL13Rα1 is associated with shortened OS and RFS (Kim et al., 2021). Next-generation sequencing (NGS)-based detection of circulating cell-free DNA (cfDNA) in sarcoma patients facilitates diagnostic refinement and longitudinal disease monitoring (Mc connell et al., 2020). Circulating tumor DNA (ctDNA) demonstrates clinical utility in tracking minimal residual disease (MRD) and early recurrence patterns (Braig et al., 2019). Thrombospondin-2 (Tsp2) encoded by THBS2 serves as an independent predictor of disease-free survival (DFS) and recurrence-free survival (RFS) in RLS cohorts (Xu et al., 2022). Fibroblast growth factor receptor substrate 2 (FRS2) exhibits high positivity rates in primary RPLS tumor specimens, demonstrating significant correlation with recurrence dynamics and survival outcomes (Chen and Miao, 2023).

## 5 Summary and prospects

**FIGURE 2:**
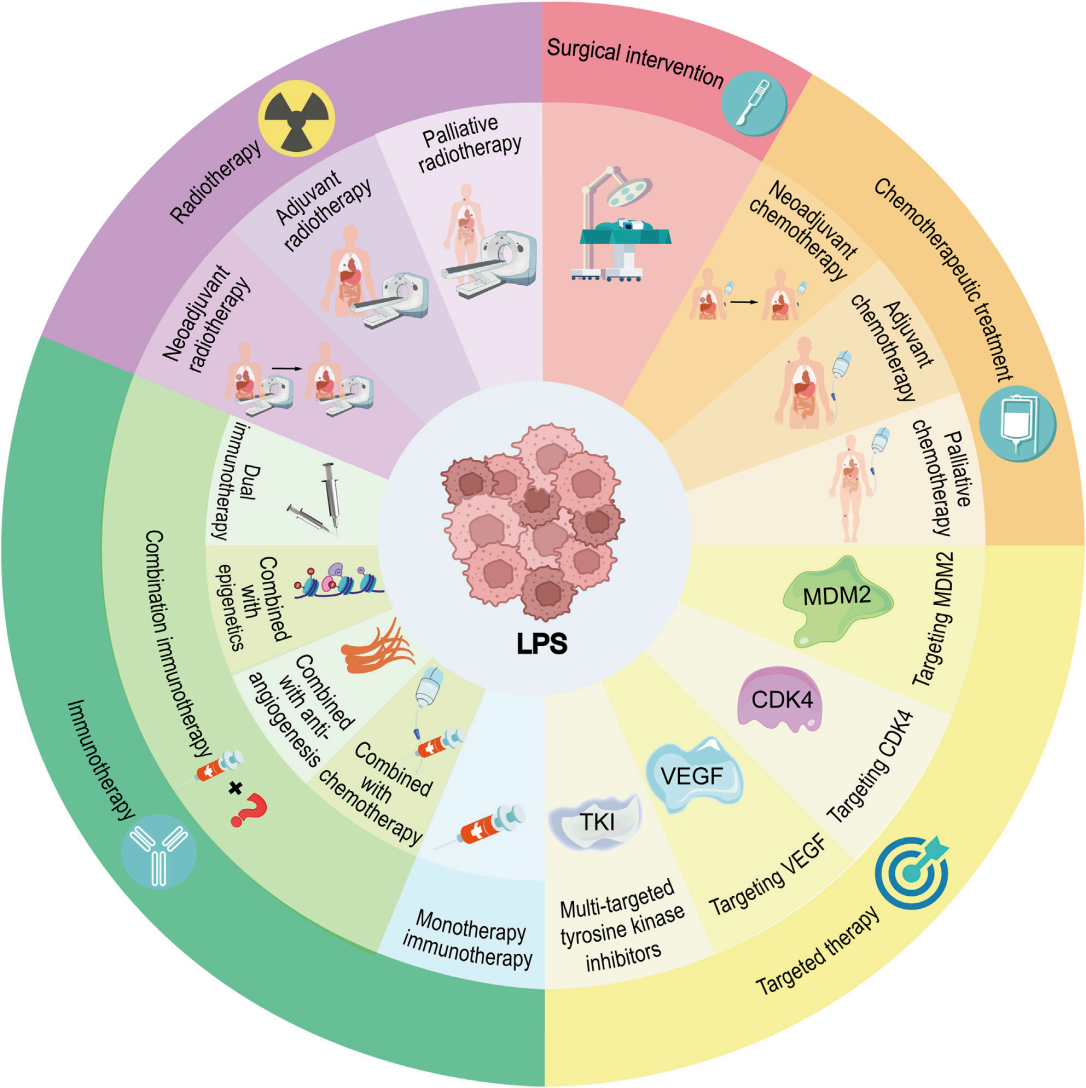
Current therapeutic landscape for liposarcoma management.

Recent research has revealed significant findings and trends in the treatment and prognosis of liposarcoma. Various treatment modalities can be seen in Figure 2. Overall, liposarcoma treatment and prognosis are influenced by various factors, including the tumor type, grade, histological characteristics, and the immune environment. Surgical resection remains the primary treatment modality for tumors such as retroperitoneal liposarcoma (RPL) and retroperitoneal sarcoma (RPS), although the risk of local recurrence after surgery is high. Advances in medical technology are expected to enhance minimally invasive surgery and precision radiotherapy, reducing treatment-related complications and side effects and thus improving patient quality of life. Adjuvant radiotherapy before and after surgery, along with novel immunotherapy, may become integral parts of treatment strategies. Preoperative radiotherapy has shown efficacy in lowering the risk of local recurrence, but the effectiveness of perioperative radiotherapy remains uncertain. Immunotherapy has exhibited potential efficacy in some studies, particularly for patients with high PD-L1 and PD-L2 expression. Future research may delve deeper into the mechanisms and efficacy of immunotherapy and identify more precise prognostic markers to personalize treatment regimens, ultimately increasing patient survival rates and quality of life.

In addition to advancements in treatment, prognosis evaluation has become more precise. By integrating various factors, such as tumor characteristics, patient factors, and treatment response, we can establish more reliable prognostic models to assist physicians and patients in making informed treatment decisions. Further research in patients with retroperitoneal liposarcoma (RPL) and retroperitoneal sarcoma (RPS) suggests that peripheral blood inflammatory markers and specific biomarkers in tumor tissue may aid in predicting early recurrence and survival rates for postoperative patients. Additionally, with a deeper understanding of tumor immunology and genomics, coupled with the ongoing development of immunotherapy and targeted therapy, personalized treatment, including targeted therapy and immunotherapy tailored to specific tumor subtypes, is poised to become a future trend. Our approach to treating liposarcoma will also become more personalized and precise. Genomic and biomarker studies will further our understanding of tumor development mechanisms and prognostic factors, providing a stronger scientific basis for personalized treatment. Furthermore, ongoing clinical trials will present opportunities for the development of novel treatment modalities and drugs, offering patients more options and improving treatment success and survival rates.

In the future, comprehensive assessment and personalized treatment plans based on multidisciplinary teams will be key to improving the prognosis of patients with liposarcoma. Additionally, conducting more large-scale clinical trials and molecular biology research is expected to provide deeper insights and breakthroughs in the treatment and prognosis of this field. Strengthening multicenter clinical research and data sharing is also a future direction to promote a comprehensive understanding of the treatment and prognostic factors of liposarcoma, accelerate the clinical translation of new treatment methods, and continuously improve treatment outcomes and survival rates for patients. With advances in science and technology and further research, we hope to find more effective treatment methods and improve the quality of life and survival of patients with liposarcoma. We anticipate a brighter future for the treatment and prognosis of liposarcoma.

Overall, significant progress has been made in the treatment and prognosis research of liposarcoma, including the exploration and application of various treatment methods, such as surgery, radiotherapy, and immunotherapy, as well as the discovery and validation of new prognostic factors. However, many challenges and unknown factors remain. The application of novel treatment methods such as personalized treatment, immunotherapy, and targeted therapy has brought new hope for patients, but further research and clinical validation are needed to determine how to select and combine these treatment options better.

In the future, we expect further in-depth research into the pathogenesis, biological characteristics, and potential of targeted therapies for liposarcoma. With the continuous development of technology, the application of high-throughput technologies such as genomics, transcriptomics, and proteomics will provide us with more comprehensive and precise tumor classification and personalized treatment strategies. Moreover, the accumulation of clinical trials and practical experience will lead to more information on the effectiveness and safety of various treatment options, helping to guide clinical practice and improve patient prognosis. In the future, with the continuous advancement of technology and research methods, we can expect more accurate diagnostic methods, more effective treatment strategies, and more accurate prognosis evaluation models to emerge. Ultimately, we hope to provide liposarcoma patients with more effective and safer treatment methods, improving their quality of life and survival rate through various efforts.
