# Supplementary material for: Challenges and hope: latest research trends in the clinical treatment and prognosis of liposarcoma
Source: Front Pharmacol. 2025 May 12;16:1529755. doi: 10.3389/fphar.2025.1529755 (PMC12104207; doi:10.3389/fphar.2025.1529755)
Supplement: Supplementary file 1 [file Table1.docx]

| STSs | Soft tissue sarcomas |
| --- | --- |
| LPS | Liposarcoma |
| ALT | Atypical lipomatous tumor |
| WDLPS | Well-differentiated liposarcoma |
| DDLPS | Dedifferentiated liposarcoma |
| MLPS | Myxoid liposarcoma |
| PLPS | Pleomorphic liposarcoma |
| MPLPS | Myxoid pleomorphic liposarcoma |
| DDIT3 | **DNA damage inducible transcript 3** |
| FUS | **Fused in sarcoma** |
| EWSR1 | EWS RNA binding protein 1 |
| MDM2 | Mousedouble minute 2 |
| CDK4 | Cyclin-dependent kinase 4 |
| HMGA2 | High mobility group AT-Hook 2 |
| TSPAN31 | Tetraspanin 31 |
| FRS2 | Fibroblast growth factor receptor substrate 2 |
| GLI1 | Glioma-associated oncogene homolog 1 |
| DDR2 | Discoidin domain receptor tyrosine kinase 2 |
| SDHC | Succinate dehydrogenase complex subunit C |
| FGFR | Fibroblast growth factor receptor |
| PIK3 | Phosphoinositide-3-kinase |
| PIK3R3 | Phosphoinositide-3-kinase regulatory subunit 3 |
| ERK | Extracellular regulated protein kinases |
| Nanog | Nanog homeobox |
| RET | Ret Proto-Oncogene |
| AKT | Protein kinase B |
| mTOR | **Mammalian target of rapamycin** |
| YAP1 | **Yes-associated protein 1** |
| TERT | Telomerase reverse tranase |
| CSC | Cancer stem cell |
| RB1 | Retinoblastoma l |
| RPL | Retroperitoneal sarcoma |
| RPLS | Retroperitoneal liposarcoma |
| OS | Overall survival |
| mOS | **Median survival time** |
| DFS | Disease-Free Survival |
| PFS | Progression-free survival |
| mPFS | **Median** progression-free survival |
| PFR | Progression-free Rate |
| ORR | Objective Response Rate |
| pCR | Pathologic Complete Response |
| DCR | Disease Control Rate |
| CR | **Complete response** |
| PR | **Partial response** |
| SD | Stable Disease |
| PD | Progressive disease |
| LR | Local recurrence |
| LRFS | Local recurrence-free survival |
| LRSM | Liver resection to treat sarcoma metastasis |
| A+I | Doxorubicin+ifosfamide |
| D+IFO | Doxorubicin+ifosfamide |
| CIPN | Chemotherapy-induced peripheral neuropathy |
| HIPEC | Hyperthermic Intraperitoneal Chemotherapy |
| HDAC2 | Histone**Deacetylase** 2 |
| VEGF | **Vascular endothelial growth factor** |
| PARP-1 | Poly (ADP-Ribose) Polymerase 1 |
| XPO1 | Exportin 1 |
| CALB1 | Calbindin 1 |
| ICIs | Immune Checkpoint Inhibitors |
| TCR-T | Engineered T cell receptor-T cell |
| TLS | Tertiary Lymphoid Structures |
| TMB | Tumor mutation burden |
| CAR-T | Chimeric**Antigen Receptor T-Cell Immunotherapy** |
| TAAs | Tumor-associated antigen |
| TME | **Tumor micro-environment** |
| TVEC | Talimogene laherparepvec |
| ARFS | Abdominal recurrence-free survival |
| MDT | Multidisciplinary**Team** |
| DSD | Disease-specific death |
| DR | Distant recurrence |
| LR | Local recurrence |
| DSS | Disease‐specific survival |
| DM | Distant metastatic |
| CCI | Crude-cumulative-incidence |
| LMS | Leiomyosarcoma |
| NLR | Neutrophil-to-lymphocyte ratio |
| PLR | Platelet Count to Lymphocyte Count Ratio |
